# Everybody's Page

**Published:** 1893-03-11

**Authors:** 


### EVERYBODY'S PAGE.

To the uninitiated there is a certain arbitrariness about the new law which Japan has seen wise to decree concerning the entire abolition of the female editing of newspapers and journals in that country. We have of late years been led to look upon the Japanese as a people in the very vanguard of social progress, but this new departure appears to be a step in the opposite line of march, and our curiosity is left unsatisfied as to whether this new law owes its existence to the faults of past women editors or to the inherent selfishness of man, who, we are told, in the future is to possess the sole birthright of the Press.

IF all the winnings from the Turf were applied in the manner that Baron de Hirsch has applied his, our hospital balance-sheets would have another story to tell. The owner of La Flèche has contributed about £42,000 of his winnings (at public stakes during 1891-1892) to various hospitals and benevolent institutions, the chief institutions which were receivers of the Baron's charity being the following: The London Hospital, £4,000; North-West London Hospital, £2,000; St. George's Hospital, £1,500; St. Mary's Hospital, £1,700; Middlesex Hospital, £2,200; University College Hospital, £1,700; West London Hospital, £1,700; Brompton Consumptive Hospital, £1,720; East London (for Children) Hospital, £1,700; Great Ormond Street (for Children) Hospital, £2,200; North-Eastern (for Children) Hospital, £1,000; Victoria (for Children) Hospital, £1,700; London Fever Hospital, £1,500; Orphanage of Mercy, Kilburn, £2,200; Royal Hospital for Children and Women, £1,200.

A TERRIBLE story, over 100 years' old, comes to us through a contemporary French newspaper about the sad consequences resultant on the successful cure of a woman who had been attacked by a violent epidemic, then prevalent in her part of the country. It appears that in the year 1773 the wife of a baker, living at Aubusson (Haute Marche), was attacked suddenly with symptoms of so grave a nature that the doctors who were called in, decided on trying extreme measures, and suggested a course of profuse perspiration as an experiment. In order to provoke this, the woman's husband bethought him of covering his wife with the fresh baked bread from out the oven. This proving effective, the sufferer was happily cured within twenty-four hours. The tragic part of the story is that the thoughtless baker imprudently sold the bread thus used, and in less than a fortnight over 200 persons had succumbed to the very illness which the same hot loaves had cured. The panic became great and widespread; and, indeed, the city gates were closed, and Intercessory prayers were used in all the churches imploring the Divine assistance in delivering citizens from the great affliction.

PAPIER-MACHÉ, or paper compressed almost to the solidity of iron is declared to be the great solid of the future. We have just heard of a new and portable hospital which is made of this compressed paper large enough for the accommodation of twenty beds. When folded up, this building (if one may so call it) condenses itself easily into a load for three transference trucks, which trucks on their part are planned to form the basis of the hospital; T-shaped joists of iron keeping their foundation steadily in place. Over this there is a flooring of compressed paper boards, which being varnished, adapt themselves to cleanliness; the walls and ceiling are formed of the same material, whilst tie beams, composed of thin wire of galvanized iron, connect the parallel walls. Ventilation is provided for by means of holes bored between the walls and the ceiling, and the windows are somewhat ingeniously made of wire gauze, with some transparent coating. It yet remains to be seen how far this portable paper hospital will adapt itself to the ever-increasing requirements of modern hygienic demands, and whether it will satisfactorily stand the test of coming out fire-proof.

THE advantage of "Light in a Sick Room" has been discussed lately in a weekly contemporary with much sound sense from the doctor's point of view, and, indeed, the salutary influence of sunshine, within doors as without, is beyond all dispute. But there is something, too, to be said on the part of the sick themselves. Why, if they were in sympathy with the medical cry for "Light, more light," should it then be necessary to lay so much stress on the necessity for this change in the condition of the rooms of home patients? The fact is that the love of darkness and quiet, which are the accompanying symptoms of most illnesses, are symptoms distinctly at variance with the dictates of modern science, which, with an unrelenting hygienic hand, floods in the full light of garish days on to the weary eyes of the sufferer. Imbued with this spirit of daylight, when recently visiting a well-known hospital, we complained of the absence of cross ventilation in one ward, but the Sister, who was both sympathetic and sensible, remarked that too much light in medical wards was certainly not conducive to the patients' comfort.

